# Domestic Summary

**Published:** 1840-02-01

**Authors:** 


### DOMESTIC SUMMARY.

### *Observations on Dextrine and Diastase.*

By William Procter, jr.

(Concluded.)

By scrutinizing the foregoing remarks, we will observe a great confusion of names, indeed almost as many sets of names as we have experimenters on the subject. There is evidently an analogy between some of the results, and the probability is, that the amidine and soluble amidin of Guérin, taken together, constitute the amidone of Payen and Persoz, as both amidine and amidone, colour blue with iodine, and both are obtained without the use of any other agent but water and temperature. The matter of amidone which causes it to swell, may be a tissue-like substance, which, when separated by ebullition, etc., constitutes the soluble amidin held in solution by amidine of Guérin.

I think we may safely differ from Raspail, who considers the dextrine by sulphuric acid of Biot, to be the soluble substance of fecula. Dextrine when obtained in that way, as well as by diastase, gives a vinous red or purple hue with iodine, while the substances which are derived directly from fecula by water give a sky blue.

Raspail says, that all, save the teguments of fecula, is soluble substance, an assertion which is not borne out by fact, for we find that amidone is not soluble.

He also asserts, that diastase is soluble gluten, or gluten held in solution by acetic acid. Wishing to test the truth of this assertion, some gluten was prepared and dissolved in acetic acid. This, then, should possess the power of diastase. Two drams of fecula were mixed with two ounces of water, and as much solution of gluten as equalled two or three grains of the undissolved substance was introduced along with it, and the temperature raised, expecting to see the reactions peculiar to diastase, but was somewhat disappointed when the mixture became gelatinous and exhibited no signs of alteration. At this crisis half a grain of diastase was added, dissolved in a very minute portion of water; the mixture in a very short time was sufficiently fluid to filter. If the acetic acid solution of gluten was prepared correctly, and there is every reason to believe that it was, this assertion of Raspail proves incorrect.

Raspail also asserts, that diastase and sulphuric acid possess no power of rupturing the envelopes of fecula, but that it is due to the water and temperature. To ascertain how far this was correct, 240 grains of fecula, about 1000 grains of water, and half a grain of diastase, were mixed together, and heat gradually applied. At the temperature of 150° Fahr., the mixture had a gelatinous consistence owing to the rupture of the envelopes, and the liberation of the interior substance. Again; the same quantities of fecula and water were mixed together *without* the diastase, and the temperature raised as before. At 150° Fahr., the mixture becomes gelatinous as in the other case. To this one-third of a grain of diastase mixed with half a dram of water was added, and the whole stirred together. In a very short time, the mixture lost its jelly character, and was quite fluid. From this it may clearly be inferred, that water and temperature, as has been said by Raspail, are *really* the agents which rupture the envelopes. It may be well to remark, that the jelly of that in which diastase was used, was not so thick as the other, which may be traced to the immediate action of the diastase on the first portion qf broken granules.

### *Preparation of Dextrine.*

First, by sulphuric acid.

The proportions recommended by different chemists are exceedingly variable, as are the processes. Berzelius directs,

| | | |
|---|---|---|
| Fecula | 50 | parts, |
| Acid | 20 | " |
| Water | 139 | " |

Mix the fecula with one-half of the water, and the acid with the other; first heat the diluted acid, and add the fecula and water, in small quantities at a time, then raise the temperature to ebullition, and keep it there a few minutes. Afterwards saturate the acid with marble dust; filter, and evaporate carefully to dryness.

Thenard* says, take one hundred parts of fecula, twenty-eight of water, and twenty of acid, which is evidently a mistake, as the quantity of water is entirely too small. The dextrine obtained by the process of Berzelius is very soluble in water, and colours vinous red with iodine. According to M. Thinus, (Journal de Pharm. tome xx,) the following are the most eligible proportions:

* Traité de Chemie, tome iv. page 371.

Fecula 50 parts.
Acid 10 "
Water 600 "

I have used these proportions in preparing it, and found them to answer very well; mix the fecula with one-half of the water, and the acid with the other, and apply heat, taking the precaution to keep the mixture in constant agitation, at 150° or 160° Fahr., the whole becomes a jelly, which gradually becomes more fluid, until the temperature arrives at 196°. Keep the mixture at this degree for some minutes, and then allow it to stand twenty-four hours, when it is to be filtered. On adding an excess of alcohol to this solution, the dextrine is precipitated in a floculent state, which aggregates in a cohesive mass. Thus obtained, it retains a small quantity of sulphuric acid, mechanically, which by re-dissolving in a small quantity of water, and again precipitating, yields the dextrine pure.

This process might be altered so as to saturate the acid with marble dust, and evaporate the solution to dryness, after filtering.

2d. A much easier, and more eligible process is that in which diastase is used as the agent. The following proportions have yielded a satisfactory result, viz:

Diastase 1 part.
Fecula 400 "
Water 1200 "

Dissolve the diastase in the water, then add the fecula, and raise the temperature gradually, stirring the mixture constantly. At 150° the whole becomes a mass of thick jelly, which liquifies entirely between 150° and 160° Fahr. When this has taken place, raise the temperature of the mixture rapidly to ebullition, which suspends the action of the diastase on the dextrine. Floating through the liquid is seen a floculent substance, which is composed of the empty teguments, upon which the diastase has no action. After the liquid has cooled, filter it, and either precipitate with alcohol, or evaporate carefully to dryness. The advantage of using the alcohol is the separation of the sugar, which invariably attends the action of diastase.

I think that we may draw from the foregoing exposition the following conclusions, viz.:

1st. That fecula consists of teguments, which envelope a peculiar substance of a spongy character, which is either one uniform matter, or composed of a soluble and an insoluble substance united.

2d. That dextrine, or gum of fecula, is always a product of art, and is derived from the interior substance of fecula, by the action of different agents.

3d. That diastase and sulphuric acid, and other agents, that are thought to exercise the power of rupturing the envelopes of fecula, do not really possess that power, but the water and temperature cause them to be ruptured, and then these agents act on the interior substance, and convert it into dextrine and sugar of amidon, thus destroying the gelatinous consistence of the mixture.

4th. That diastase is not soluble gluten, as asserted by Raspail, but is a substance enjoying the power of converting the interior substance of fecula, first into dextrine, and afterwards into sugar of amidon.—*Am. Journ. Pharm., Jan.* 1840.

